# Increased catalytic activity through ZnMo_7_O_24_/g-C_3_N_4_ heterostructured assemblies for greener indole condensation reaction at room temperature

**DOI:** 10.1038/s41598-022-23447-8

**Published:** 2022-11-03

**Authors:** Najmedin Azizi, Elham Farhadi, Fezeh Farzaneh

**Affiliations:** Chemistry & Chemical Engineering Research Center of Iran, P.O. Box 14335-186, Tehran, Iran

**Keywords:** Environmental sciences, Solid Earth sciences, Chemistry

## Abstract

As an economical conjugated polymer, graphitic carbon nitride (g-C_3_N_4_) has recently attracted much attention due to its exciting chemical and thermal stability and easy availability. Herein, we constructed a metal-coordinated graphitic carbon nitride (M–g-C_3_N_4_) catalyst through simple impregnation and calcination methods and used it as a new heterogeneous catalyst for the efficient synthesis of bis (indolyl) methanes and trisindolines under mild conditions. This reaction is performed efficiently in water as an environmentally friendly solvent at ambient conditions. The ZnMo_7_O_24_/g-C_3_N_4_ nanocomposite was synthesized by a simple method by immobilizing Mo_7_O_24_(NH_4_)_6_·4H_2_O and ZnCl_2_ on the surface of g-C_3_N_4_ under hydrothermal conditions. It was characterized by FT-IR, EDS, and electronic scanning microscopy (SEM). The metal doping of Mo and Zn on the surface of graphitic carbon nitride leads to the formation of a green catalyst that gives good to excellent yields of products in short reaction times with an easy working procedure. In addition, the ZnMo_7_O_24_/g-C_3_N_4_ catalyst could be reused at least five runs without apparent loss of efficiency.

## Introduction

The indole derivatives are important nitrogen-containing compounds due to their diverse pharmacological activities^1,2^. The indole alkaloids^3,4^, from lysergic^5^ acid to vincristine^6^ are one of the largest classes of alkaloids^7^, and they possess extended biological activity and drug discovery^8^. Among various reactions of indole^9,10^, the condensation reactions of indole with electron-deficient carbonyl compounds for the preparation of bis (indolyl) methanes and trisindolines has attracted and continues to attract interest in recent years^11,12^. In this context, various articles have focused on the preparation of target compounds employing homo and heterogeneous catalysts such as acidic ionic liquid immobilized on silica^13^, LiClO_4_^14^, silica sulfuric acid^15^, magnetic metal–organic framework^16^, graphene^17^, Protic solvents^18^ and heteropoly acids^19^. Although these methods have some advantages, most have fundamental weaknesses, such as harsh reaction conditions, volatile organic solvents, toxic reagents and solvents, limited substrate scope, expensive reagents, and catalyst overload. In recent years, the literature has also documented various green protocols, such as organocatalyst^20,21^, ionic liquids^22^, deep eutectic solvents^23^, ultrasounds^24^, and Taurine^25^ for the efficient synthesis of indole derivatives.

Carbon nanomaterials have become a new research hotspot in sensors, drug delivery, photocatalysis, and energy-saving^26,27^. Graphite carbon nitrides (g-C_3_N_4_) as a fascinating conjugated polymer constructed from two-dimensional sheets with outstanding potential for catalytic and optoelectronic applications. Its physicochemical properties, such as resistance to acidic or basic media, extended chemical, and thermal stability, fascinating electronic properties, and unique structure, have elicited interdisciplinary research fascination^28,29^. g-C_3_N_4_ consists of earth-abundant carbon and nitrogen elements with a high degree of density and is the most stable allotrope of carbon nitrides in the ambient atmosphere^30^. It has rich surface properties due to its many nitrogen coordination sites suitable for catalytic applications^31–34^. In addition, many free amino groups on the C_3_N_4_ backbone made these compounds rich in electron lone pairs easily bound to metal ions^25^, doping g-C_3_N_4_ with metal and nonmetal ions showed significant improvement in their catalytic activity^35–37^. Furthermore, graphitic carbon nitride can easily be obtained under solid-state conditions without organic solvents^38,39^ from inexpensive materials such as melamine or urea derivatives^40^.

In continuation of our research by using green solvents and catalysts in organic transformations^41,42^ herein, we have reported a simple, mild, and general method for synthesizing indole derivatives in water in the presence of ZnMo_7_O_24_/g-C_3_N_4_ as a new separable and inexpensive heterogeneous composite.

## Experimental

### General

All chemicals, such as aldehydes, indole, ketones, isatin, Mo_7_O_24_(NH_4_)_6_·4H_2_O, and ZnCl_2_ were commercially available and used without further purifications. Solvents were purchased from commercial sources and distilled before use. The Buchi Melting point M-535 is used to determine melting temperatures.

### Preparation of ZnMo_7_O_24_/g-C_3_N_4_

**Figure 1:**
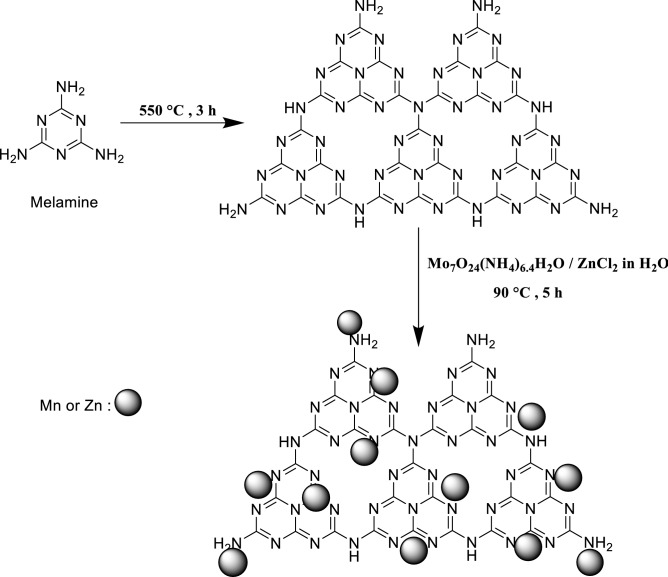
Synthesis of ZnMo_7_O_24_/g-C_3_N_4_.

The bulk g-C_3_N_4_ was prepared by thermal polymerization of melamine according to the reported procedure^38^. In detail, 20 g of melamine in 150 mL crucible is heated to 550 °C with a heating rate of 5 °C min^−1^ and kept at 550 °C for 3 h in an air atmosphere. The resultant light yellow agglomerates was ground by an agate mortar for the next steps. The ZnMo_7_O_24_/g-C_3_N_4_ composites were prepared by a facile chemical method. 0.5 g of g-C_3_N_4_ was dispersed in 50 mL deionized water using a stirrer for 10 min at room temperature. In the next step, 0.2 g of Mo_7_O_24_(NH_4_)_6_.4H_2_O was dispersed in 20 mL of deionized water under stirring. In another flask, 0.2 g of ZnCl_2_ was added to 20 mL of deionized water and dissolved by magnetic stirring. Then, Mo_7_O_24_(NH_4_)_6_.4H_2_O and ZnCl_2_ solutions were added to the g-C_3_N_4_ suspension, and a magnetic stirrer was used to stir the reaction mixture for 5 h at 90 °C. After completion of the reaction, the solvent was removed in a vacuum by rotary evaporator and was dried at room temperature for 12 h, and Blue-green powder ZnMo_7_O_24_/g-C_3_N_4_ catalysts were obtained (Fig. 1).

### General procedure for the synthesis of bis‐indoles

Indole (1.0 mmol), aldehyde (0.5 mmol), and ZnMo_7_O_24_/g-C_3_N_4_ (15 mg) in deionized water (1.0 mL) were stirred well using a magnetic stirrer, and TLC assessed the progress of the reaction until the reaction completion. Then, ethyl acetate (10 mL) and water (10 mL) were added to the reaction mixture and centrifuged. The organic phase was removed under reduced pressure, and the crude product was purified by recrystallization in ethanol, ethyl acetate, or column chromatography to afford the corresponding products. All products were known and identified by melting point.

### General procedure for the synthesis of trisindolines

A mixture of indole (1.0 mmol), isatin (0.5 mmol), and ZnMo_7_O_24_/g-C_3_N_4_ (30 mg) in deionized water (1.0 mL) conditions was stirred at room temperature, and TLC tracked the reaction progress. After completion, the reaction mixture was diluted with water and ethyl acetate and centrifuged to give the crude product after evaporation of ethyl acetate. The crude product was purified by silica gel column chromatography or recrystallized in ethanol or ethyl acetate to afford the corresponding pure trisindolines (Supplementary Information).

## Results and discussion

The co-condensation procedure was used to synthesize pure g-C_3_N_4_. The ZnMo_7_O_24_/g-C_3_N_4_ nanocomposite was synthesized by immobilizing Mo_7_O_24_(NH_4_)_6_.4H_2_O and ZnCl_2_ on the surface of g-C_3_N_4_. The morphology and structure of nanocomposite were thoroughly characterized by X-ray diffraction (XRD), scanning electron microscopy (SEM), and Fourier transform infrared spectroscopy (FT-IR).

**Figure 2:**
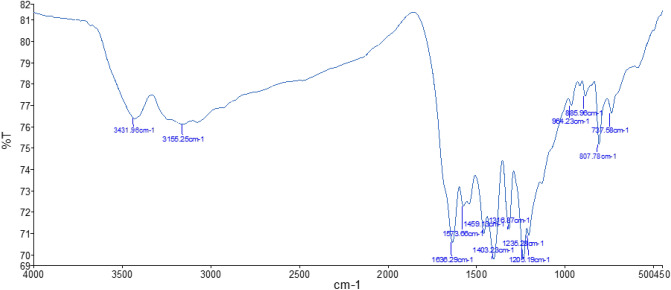
FT-IR spectra of ZnMo_7_O_24_/g-C_3_N_4_.

FTIR analysis was further carried out to identify the functional groups, and the results are shown in Fig. 2. The absorption peak at 3000–3500 cm^−1^ is related to the stretching vibration of NH and NH_2_ groups in the g-C_3_N_4_ or adsorb water from the environment. The prominent characteristic peaks in the area 1636, 1573, 1403, 1317, and 1235 cm^−1^ represent the stretching vibrations of s-triazine or tri-s-triazine of g-C_3_N_4_ in the sample. Besides, the strong absorption peak at 807 cm^−1^ is the bending vibration of the s-triazine rings system.

**Figure 3:**
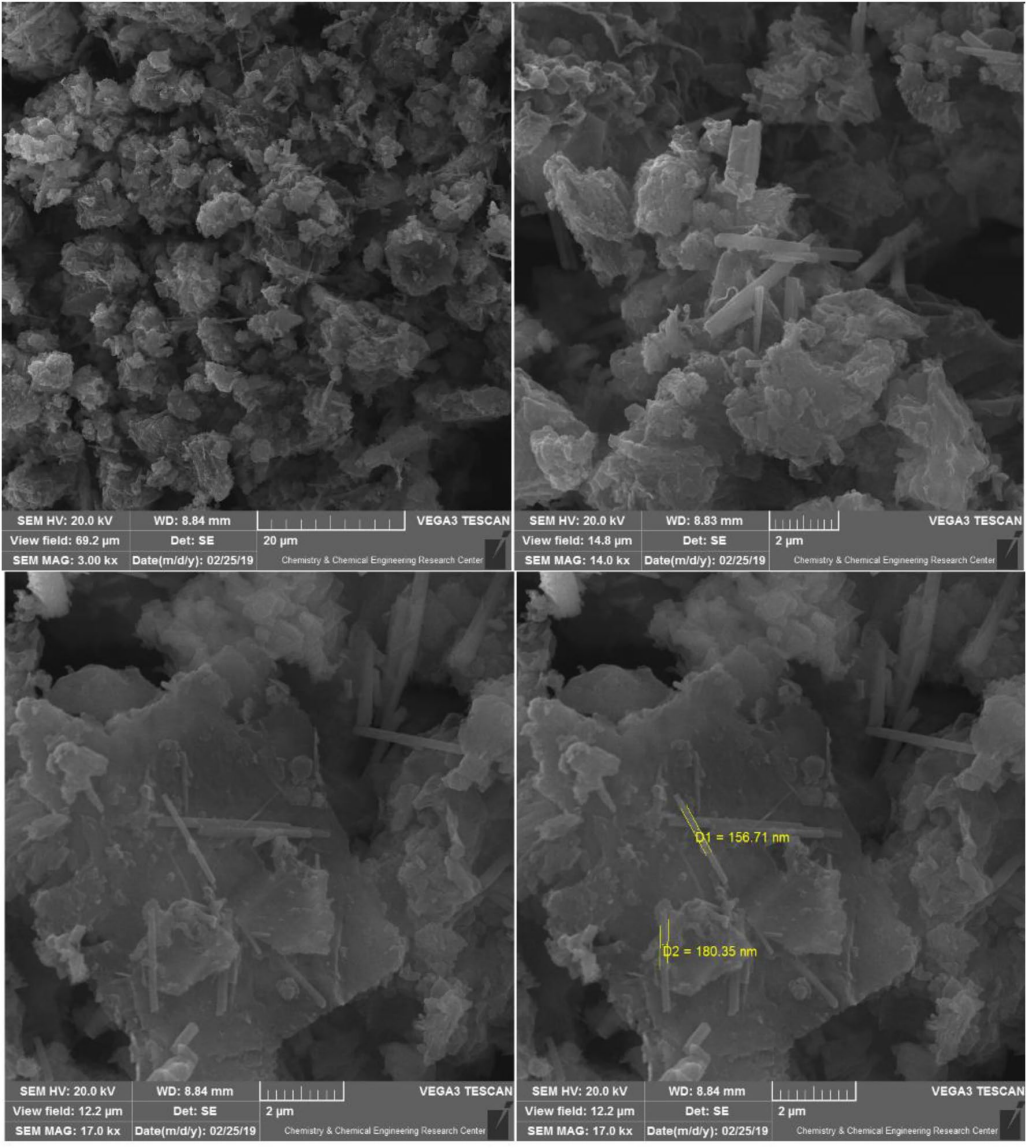
SEM images of ZnMo_7_O_24_/g-C_3_N_4_.

The typical SEM microscopy analysis is presented in Fig. 3 to investigate the new nanocomposite's morphology. The SEM spectrum of the ZnMo_7_O_24_/g-C_3_N_4_ catalyst indicates a series of thin sheets with wrinkles and irregular folding structures on the surface of g-C_3_N_4_.

**Figure 4:**
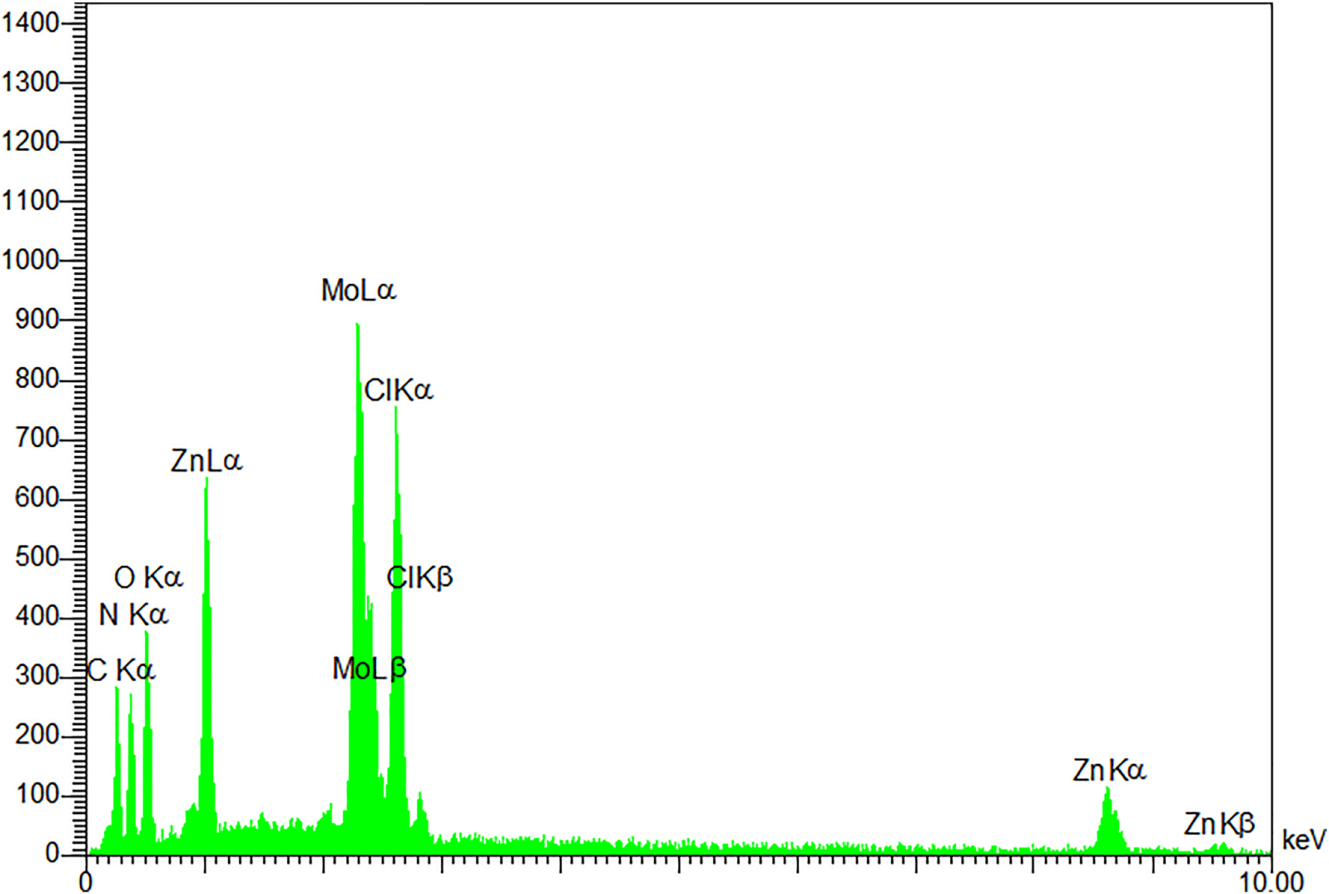
EDS spectrum of ZnMo_7_O_24_/g-C_3_N_4_.

The energy dispersive spectroscopy (EDS) technique is used for the qualitative analysis of ZnMo_7_O_24_/g-C_3_N_4_. This pattern showed the Mo, Zn, and Cl elements are identified beside the C and N elements. As shown in Fig. 4, adopting Mo and Zn nanoparticles onto the g-C_3_N_4_ was efficacious. Also, the spectrum reveals that the scattering of these nanoparticles on the g-C_3_N_4_ substrates is uniform and acceptable.

After preparation and characterization of the ZnMo_7_O_24_/g-C_3_N_4_ composite, the catalytic activity of the composite was evaluated in the preparation of bis (indolyl) methanes via the reaction 2-methylindole (1.0 mmol) and aldehyde (0.5 mmol) in the presence of ZnMo_7_O_24_/g-C_3_N_4_ as a catalyst in deionized water (1.0 mL) to optimize reaction parameters (Table 1). The greener synthesis of bis (indolyl) methanes was carried out in a 5 mL three-necked round flask equipped with a magnetic stirrer, and the mixture was vigorously stirred at room temperature.

**Table 1:** Optimization of the synthesis of bis (indolyl) methanes.


Entry	ZnMo_7_O_24_/g-C_3_N_4_ (mg)	Solvents (1 mL)	Yields (%)^a^
1	5	Water	67
2	10	Water	86
3	15	Water	95
4	20	Water	95
5	15	Ethanol	84
6	15	DMF	76
7	15	THF	72
8	15	Toluene	45
9	15	Ethyl acetate	44
10	15	CH_3_CN	56
11 ^b^	15	Water	32
12 ^c^	15	Water	75
13^d^	15	Water	57
14^e^	15	Water	–
15^f^	–	Water	–

^a^Isolated yields.

^b^Na_2_Mo_7_O_24_.

^c^ZnMo_7_O_24_ as a catalyst.

^d^ZnCl_2_ catalyst.

^e^g-C_3_N_4_ as a catalyst.

^f^Without catalyst.

The first finding indicated that the synthesis of bis(indolyl)methanes in the presence of the ZnMo_7_O_24_/g-C_3_N_4_ (15 mg) in deionized water (1.0 mL) was accomplished within 120 min with quantitative yields. (Table 1, entry 3). First, the amount of ZnMo_7_O_24_/g-C_3_N_4_ on the model reaction was optimized, and the results are shown in Table 1. The maximum yields of 95% were obtained when the loaded amount of composite was 15 mg (Table 1, entry 3). As the loaded amounts of composite increased to 20 mg, the reaction yields did not increase (Table 1, entry 4). While the amount of composite is reduced to 5 and 10 mg, increased reaction time was needed to achieve the optimal results (Table 1, entries 1–2). Furthermore, composite elements such as g-C_3_N_4_ (Table 1, entry 14) and ZnMo_7_O_24_ (Table 1, entry 12) Na_2_Mo_7_O_24_ (Table 1, entry 11) ZnCl_2_ (Table 1, entry 13) gave reduced yields. The model reaction was performed in different polar and nonpolar solvents (Table 1, entries 5–10) to optimize reaction conditions. The model reaction in organic solvents such as ethanol, dimethylformamide, and tetrahydrofuran in the presence of ZnMo_7_O_24_/g-C_3_N_4_ (15 mg) formed the expected product in lower yield.

The general nature of the procedure was confirmed by using structurally various aromatic and aliphatic aldehydes bearing electron-withdrawing and electron-donating substituents in the reaction with indole derivatives under the optimized conditions (Table 2). As seen in Table 2, different electron-donating or electron-withdrawing groups in the benzaldehyde ring proceeded well with 2-methylindole or indol, which gives good to excellent yields under short reaction times. It is necessary to mention that no remarkable reactivity differences were observed. In other words, the aromatic aldehydes with the electron-donating groups increased the yield slightly. They gave well to excellent results, while electron-withdrawing benzaldehyde derivatives did not reduce the reactivity. As an exception, the interaction of 2-hydroxy benzaldehyde and 2-methylindole yielded a lower yield (70%) than the other aldehydes. In addition, cyclohexanone generated the corresponding product in only a moderate yield under identical reaction conditions.

**Table 2:** The synthesis of bis (indolyl) methanes using ZnMo_7_O_24_/g-C_3_N_4_ as the catalyst.


Entry	Aldehydes (R-CHO)	Indole	Products	M.P. (°C)	Yields (%)^a^
1	C_6_H_5_-	2-Methylindole	**3a**	120–121 118–120^13^	95
2	C_6_H_5_-	1-Methylindole	**3b**	182–184 181–183^13^	96
3	3-OMe-C_6_H_4_	2-Methylindole	**3c**	237–239 236–238^15^	93
4	4-Cl-C_6_H_4_	2-Methylindole	**3d**	229–230 228–230^14^	90
5	4-Me-C_6_H_4_	2-Methylindole	**3e**	217–218 217–219^14^	95
6	4-OMe-C_6_H_4_	2-Methylindole	**3f.**	194–195 194–196^14^	79
7	2,4-Cl-C_6_H_3_	1-Methylindole	**3 g**	136–138 210–212^14^	94
8	Thiophene-2-	1-Methylindole	**3 h**	147–149 148–150^19^	89
9	4-NO_2_-C_6_H_4_	2-Methylindole	**3i**	238–241 240–242^19^	91
10	C_6_H_5_-	Indole	**3j**	52–53 52–53^17^	95
11	4-Me-C_6_H_4_	Indole	**3 k**	219–221 220–221^17^	95
12	3-NO_2_-C_6_H_4_	Indole	**3 l**	214–216 216–218^19^	85
13	2,4-Cl-C_6_H_3_	Indole	**3 m**	103–105 103–106^18^	94
14	4-CO_2_Me-C_6_H_4_	Indole	**3n**	219–221 219–221^19^	91
15	Cyclohexanone	Indole	**3o**	184–186 184–187^14^	73

^a^Isolated yields.

**Figure 5:**
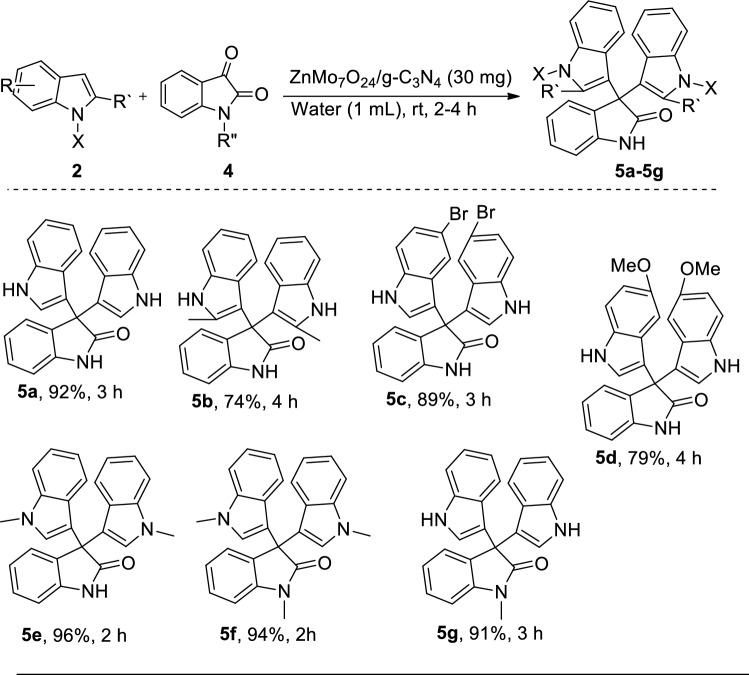
The synthesis of trisindoline using ZnMo7O24/g-C3N4 as the catalyst.

The heterocyclic spirooxindole skeleton, such as isatin containing core structure, has different biological activities and can function as synthons for naturally occurring alkaloids and pharmaceutically important drug molecules^15,16^. Encouraged by this success, we extended this reaction of substituted isatin with indole derivatives to obtain trisindoline compounds with ZnMo_7_O_24_/g-C_3_N_4_ as the catalyst (Fig. 5). Initially, indole (1.0 mmol) and isatin (0.5 mmol) reacted in the presence of ZnMo_7_O_24_/g-C_3_N_4_ as a catalyst in deionized water (1.0 mL). The results showed that 30 mg of catalyst at room temperature provided the optimum yield (92%) for the corresponding trisindoline within 180 min. The reaction of isatins and different indoles containing electron-donating and electron-withdrawing group substituent on nitrogen proceeded smoothly with good to excellent yields in 2.5–4 h.

**Figure 6:**
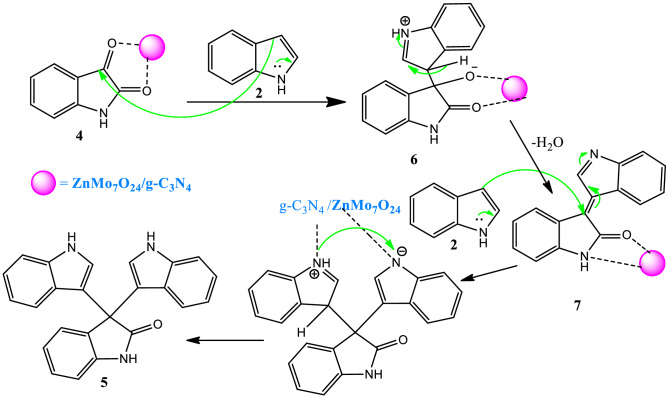
The possible reaction mechanism between isatin and indole for the synthesis of trisindoline catalyzed by ZnMo_7_O_24_/g-C_3_N_4_.

Figure 6 shows the possible catalytic pathway for the ZnMo_7_O_24_/g-C_3_N_4_ catalyzed the synthesis of trisindoline. A Zn or Mo Lewis acid coordinates to carbonyl groups of Isatin **4,** and the nucleophilic attack of indole **2** to activated carbonyls **4** creates the zwitterionic species **6**. The resulting intermediate **6** undergoes dehydration to provide the coordinated intermediate **7**, which can be captured by the second addition of indole **2** to furnish target product **5**. We proposed porous graphitic carbon nitride (g-C_3_N_4_)-stabilized ZnMo_7_O_24_ materials as in protic solvents leading to highly organodispersible and colloidally stable carbon nitrides as bifunctional Lewis acid composite for condensation reaction.

**Figure 7:**
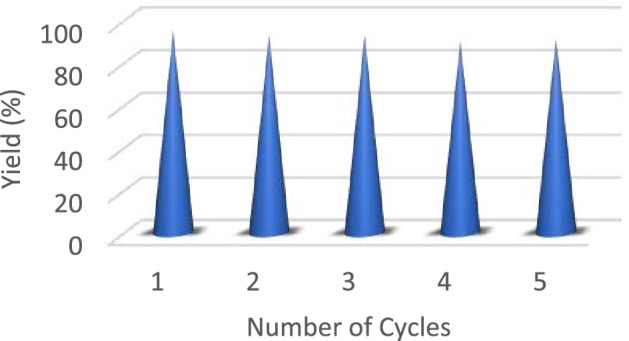
Recycling results of the ZnMo_7_O_24_/g-C_3_N_4_ for the synthesis of trisindoline.

An imperative topic for implementing a heterogeneous composite is its recovery and reusability. The ZnMo_7_O_24_/g-C_3_N_4_ catalyst could be separated by centrifugation after each run. To show the recyclability of the ZnMo_7_O_24_/g-C_3_N_4,_ the composite was recycled five times, and the results are shown in Fig. 7. Figure 4 shows the corresponding yields of the reused composite for a **5a**, which demonstrates that the catalytic activity of ZnMo_7_O_24_/g-C_3_N_4_ did not significantly decrease after being used five times. After the reaction completion, ethyl acetate was added, and the reaction mixture was centrifuged and dried under a vacuum, and used for the next cycle. The SEM and FTIR images of the reused composite after 5 cycles did not change the nanocomposite morphology.

## Conclusion

In this study, we have reported a simple and efficient method for the synthesis of bis (indolyl) methanes and trisindolines derivatives using a novel heterogeneous catalyst, Mo_7_O_24_(NH_4_)_6_·4H_2_O and ZnCl_2_ supported on graphitic carbon nitride (g-C_3_N_4_). The outstanding features of this catalyst were good to excellent yield, short reaction times, simple separation, and easy work-up. The g-C_3_N_4_ is considered an inexpensive and high surface area support for synthesizing bis (indolyl) methanes and trisindolines derivatives. Also, the ZnMo_7_O_24_/g-C_3_N_4_ showed high stability and reusability over several reaction sets without significant catalytic activity and selectivity loss.

## Supplementary Information

Supplementary Information.

## Data Availability

The data that support the findings of this study are available on request from the corresponding author.
