# Supplementary material for: Increased catalytic activity through ZnMo7O24/g-C3N4 heterostructured assemblies for greener indole condensation reaction at room temperature
Source: Sci Rep. 2022 Nov 3;12:18634. doi: 10.1038/s41598-022-23447-8 (PMC9633728; doi:10.1038/s41598-022-23447-8)
Supplement: Supplementary file 1 — Supplementary Information. [file 41598_2022_23447_MOESM1_ESM.pdf]

# Increased catalytic activity through $ZnMo_7O_{24}/g\text{-}C_3N_4$ heterostructured assemblies for greener indole condensation reaction at room temperature

*Najmedin Azizi (PhD)*, Elham Farhadi, Fezeh Farzaneh (PhD)*

Chemistry & Chemical Engineering Research Center of Iran, P.O. Box 14335-186, Tehran, Iran

Fax: +98-21-44580-775.

E-mail: azizi@ccerci.ac.ir.

**General procedure for the synthesis of bis-indoles**

Indole (1.0 mmol), aldehyde (0.5 mmol), and $ZnMo_7O_{24}/g\text{-}C_3N_4$ (15 mg) in deionized water (1.0 mL) were stirred well using a magnetic stirrer, and TLC assessed the progress of the reaction until the reaction completion. Then, ethyl acetate (10 mL) and water (10 mL) were added to the reaction mixture and centrifuged. The organic phase was removed under reduced pressure, and the crude product was purified by recrystallization in ethanol, ethyl acetate, or column chromatography to afford the corresponding products. All products were known and identified by melting point.

**General procedure for the synthesis of trisindolines**

A mixture of indole (1.0 mmol), isatin (0.5 mmol), and $ZnMo_7O_{24}/g\text{-}C_3N_4$ (30 mg) in deionized water (1.0 mL) conditions was stirred at room temperature, and TLC tracked the reaction progress. After completion, the reaction mixture was diluted with water and ethyl acetate and centrifuged to give the crude product after evaporation of ethyl acetate. The crude product was purified by silica gel column chromatography or recrystallized in ethanol or ethyl acetate to afford the corresponding pure trisindolines.

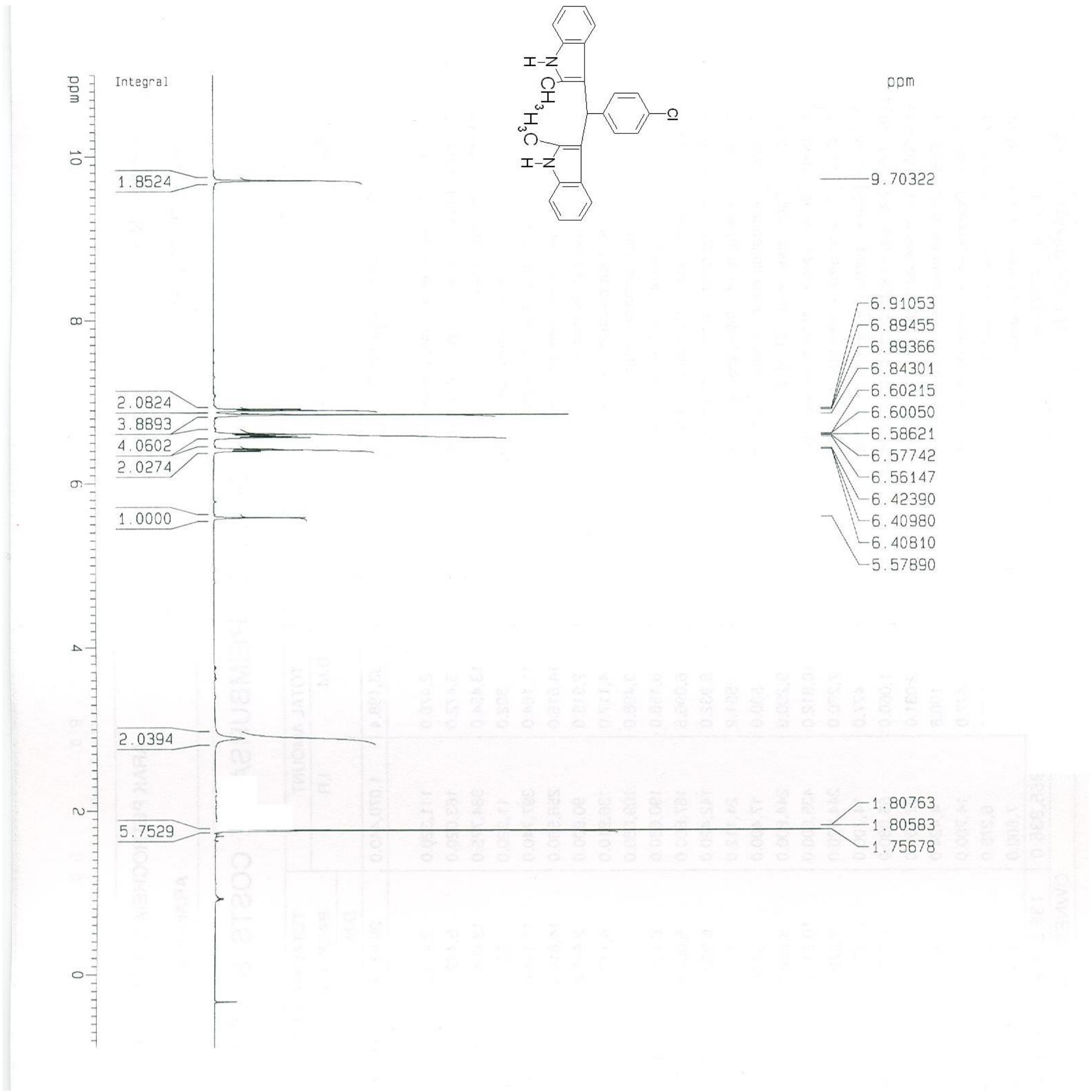


*Figure S 1. $^{1}$H NMR spectra of compound* **3d** *(Table 2, Entry 4).*

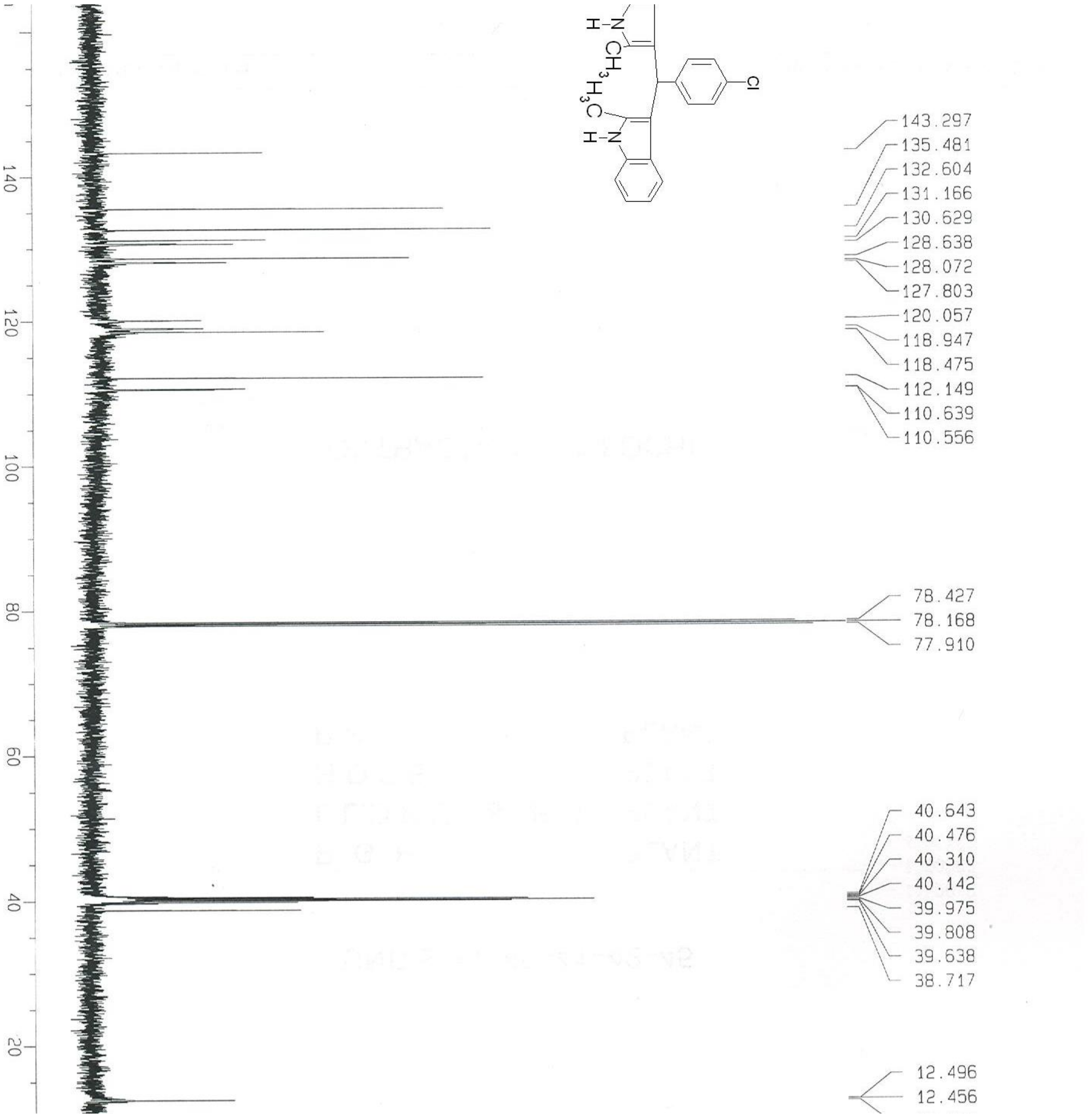


*Figure S 2. $^{13}$C NMR spectra of compound 3d (Table 2, Entry 4).*

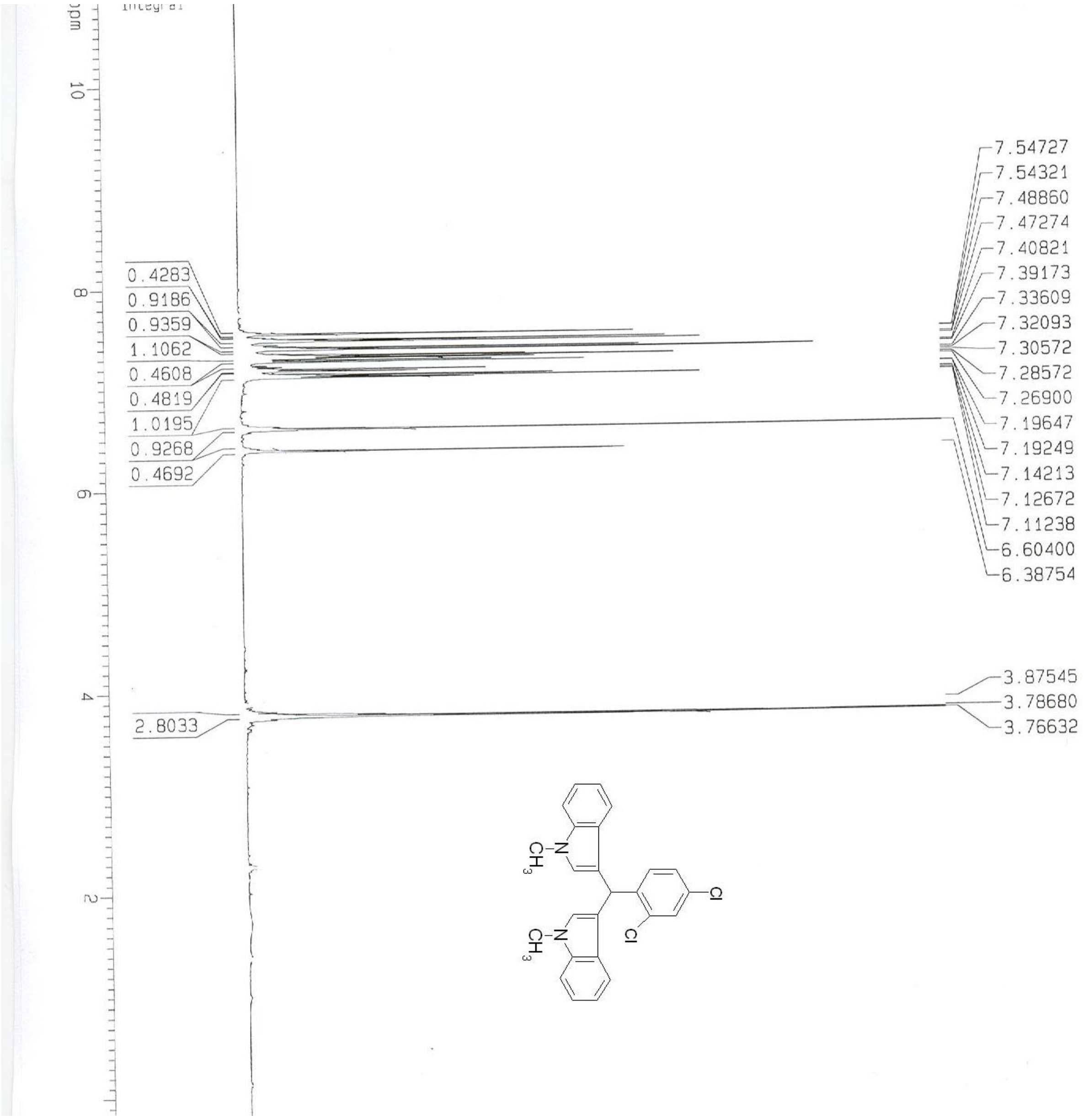


*Figure S 3. $^{1}$H NMR spectra of compound* ***3g*** *(Table 2, Entry 7).*

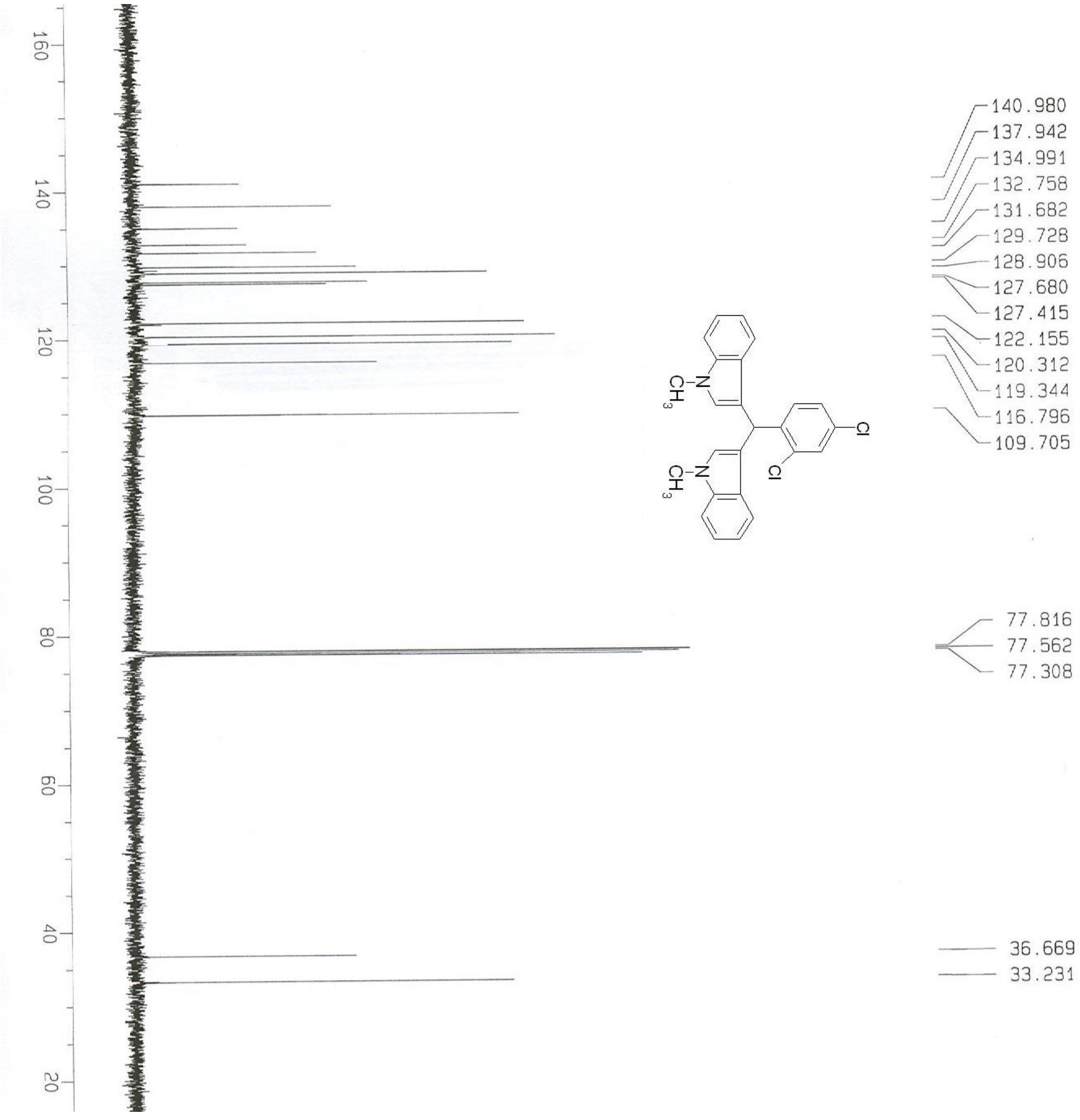


*Figure S 4. ${}^{13}$C NMR spectra of compound **3g** (Table 2, Entry 7).*

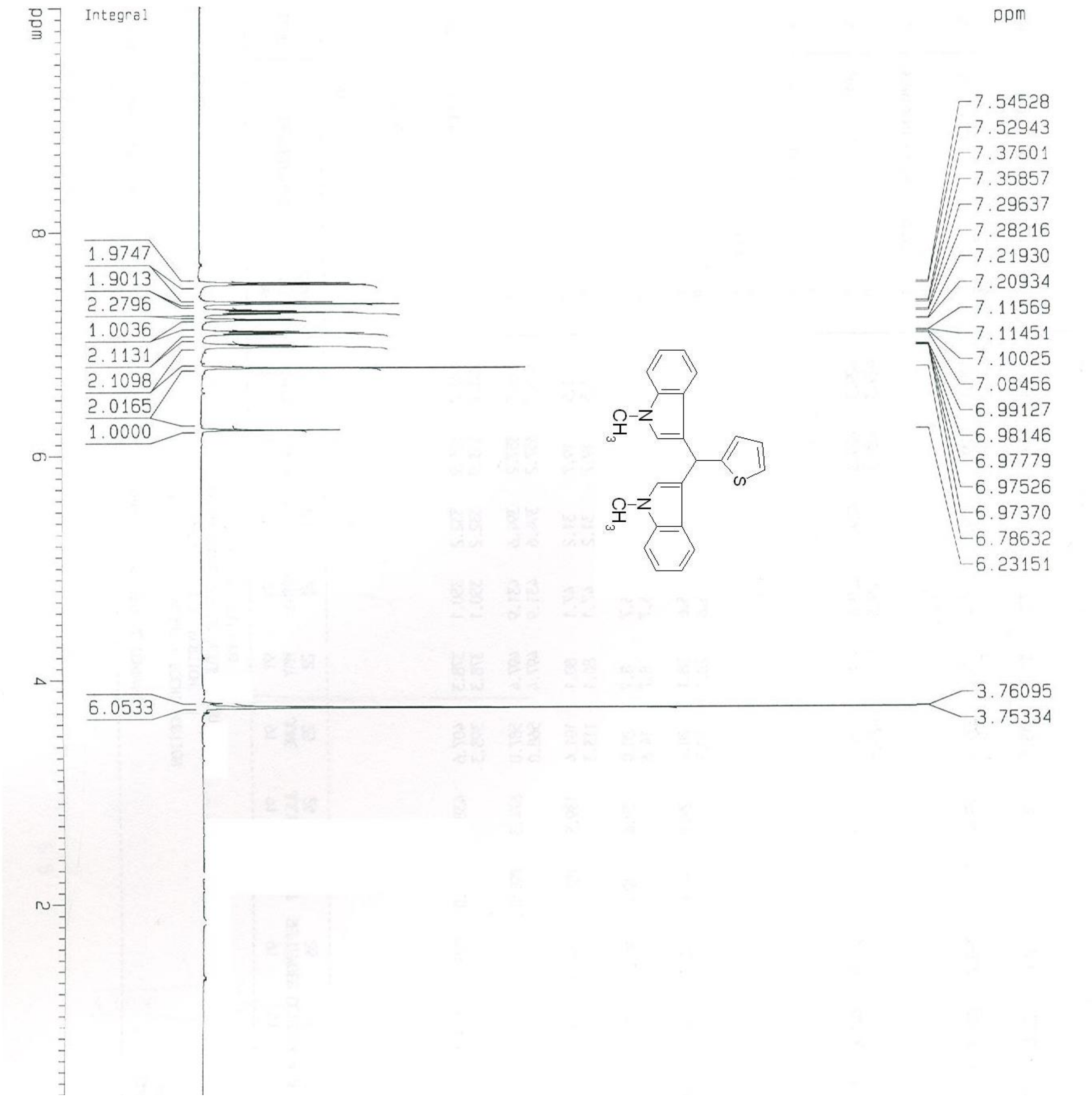


*Figure S 5. $^{1}$H NMR spectra of compound **3h** (Table 2, Entry 8).*

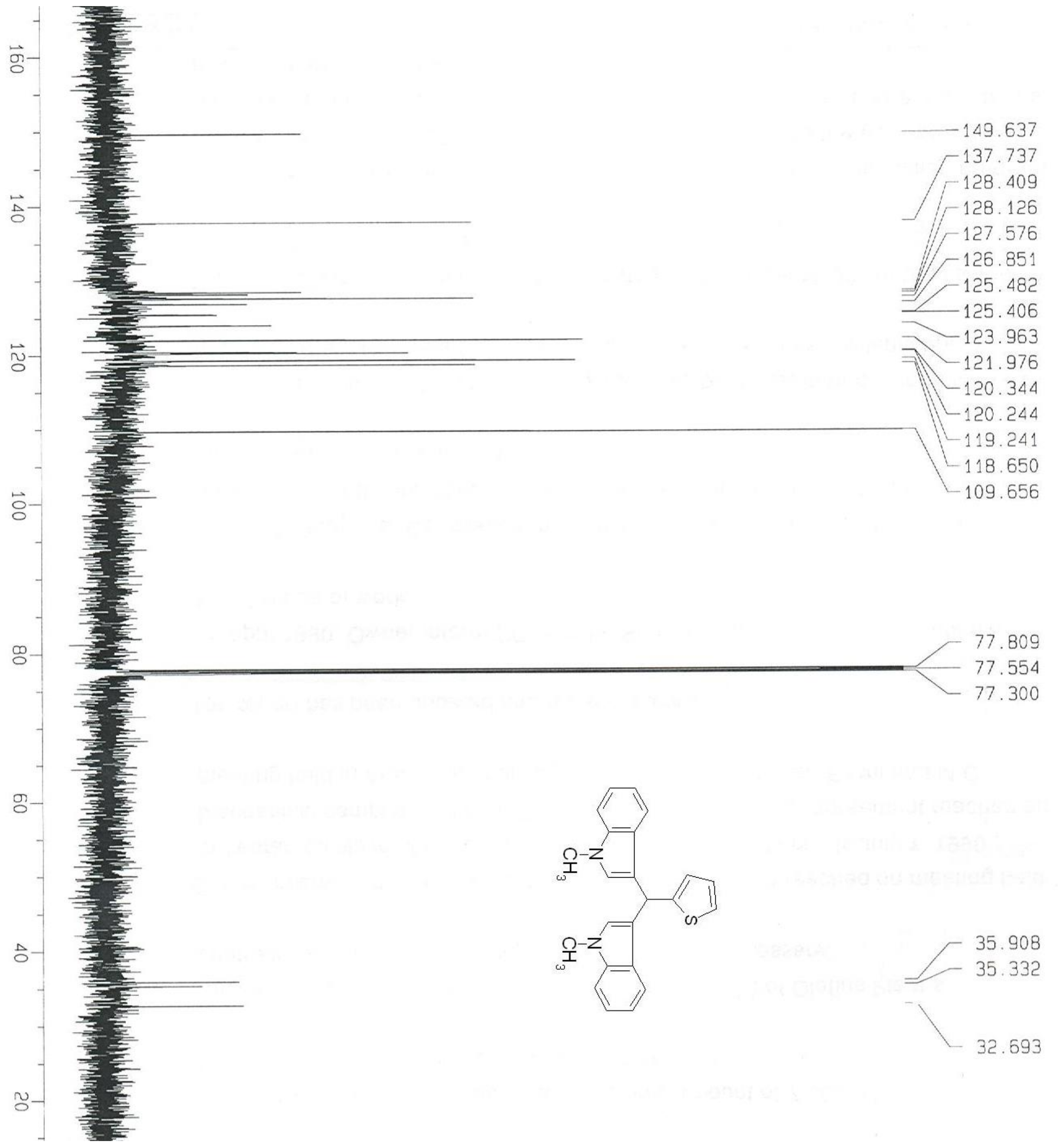


*Figure S 6.* $^{13}$*C NMR spectra of compound* ***3h*** *(Table 2, Entry 8).*

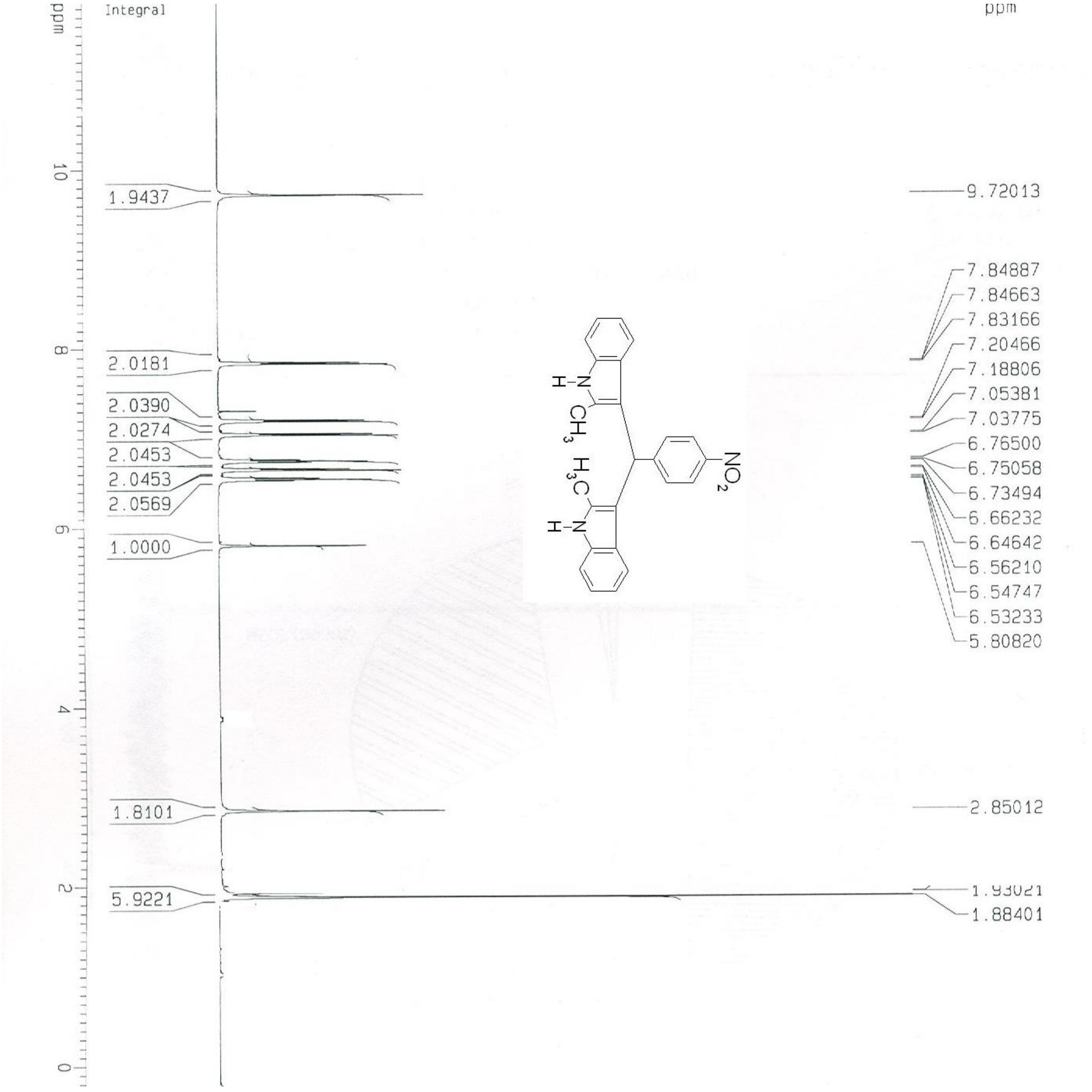


*Figure S 7. $^{1}$H NMR spectra of compound **3i** (Table 2, Entry 9).*

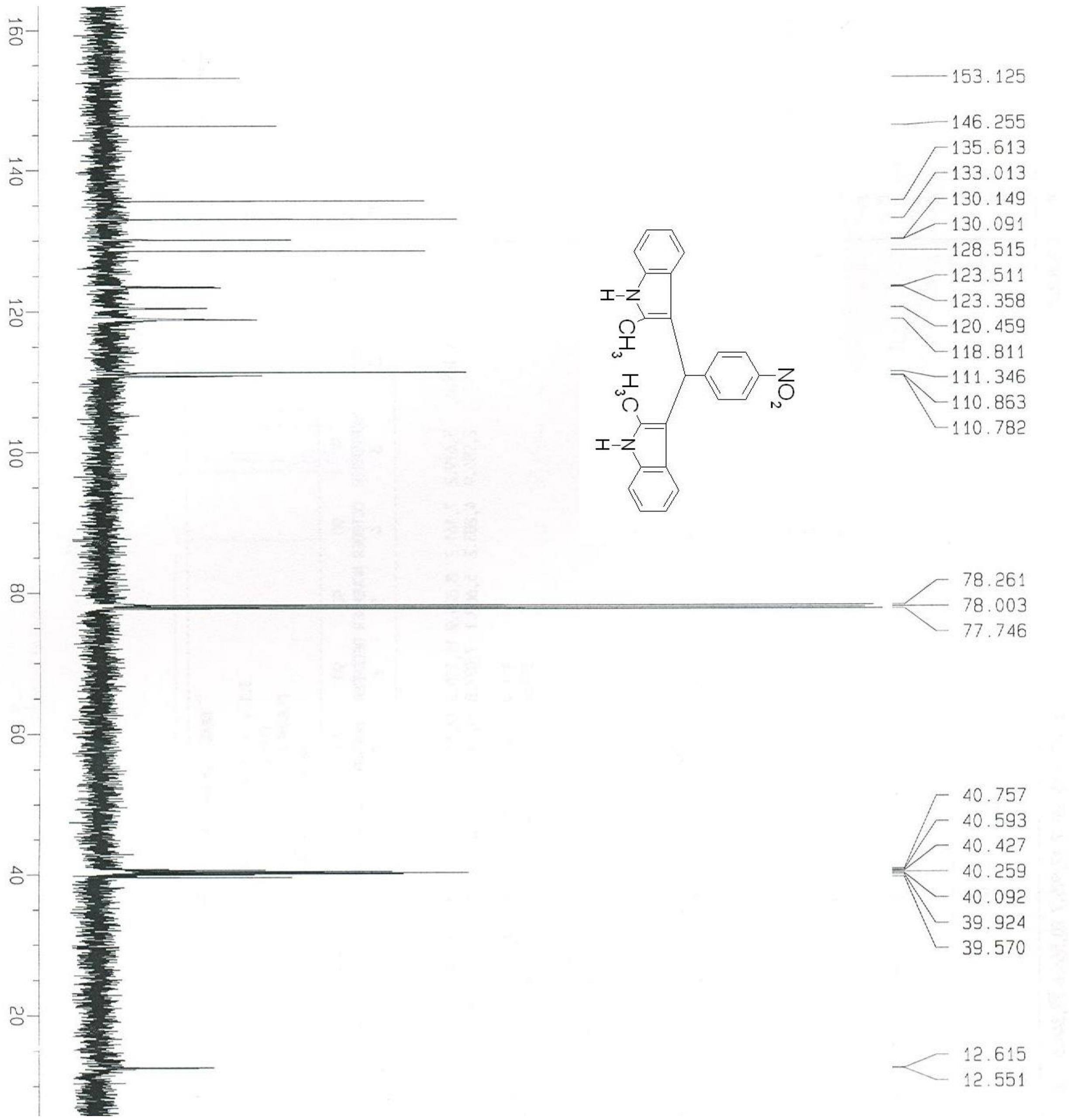


*Figure S 8. $^{13}$C NMR spectra of compound* ***3i*** *(Table 2, Entry 9).*

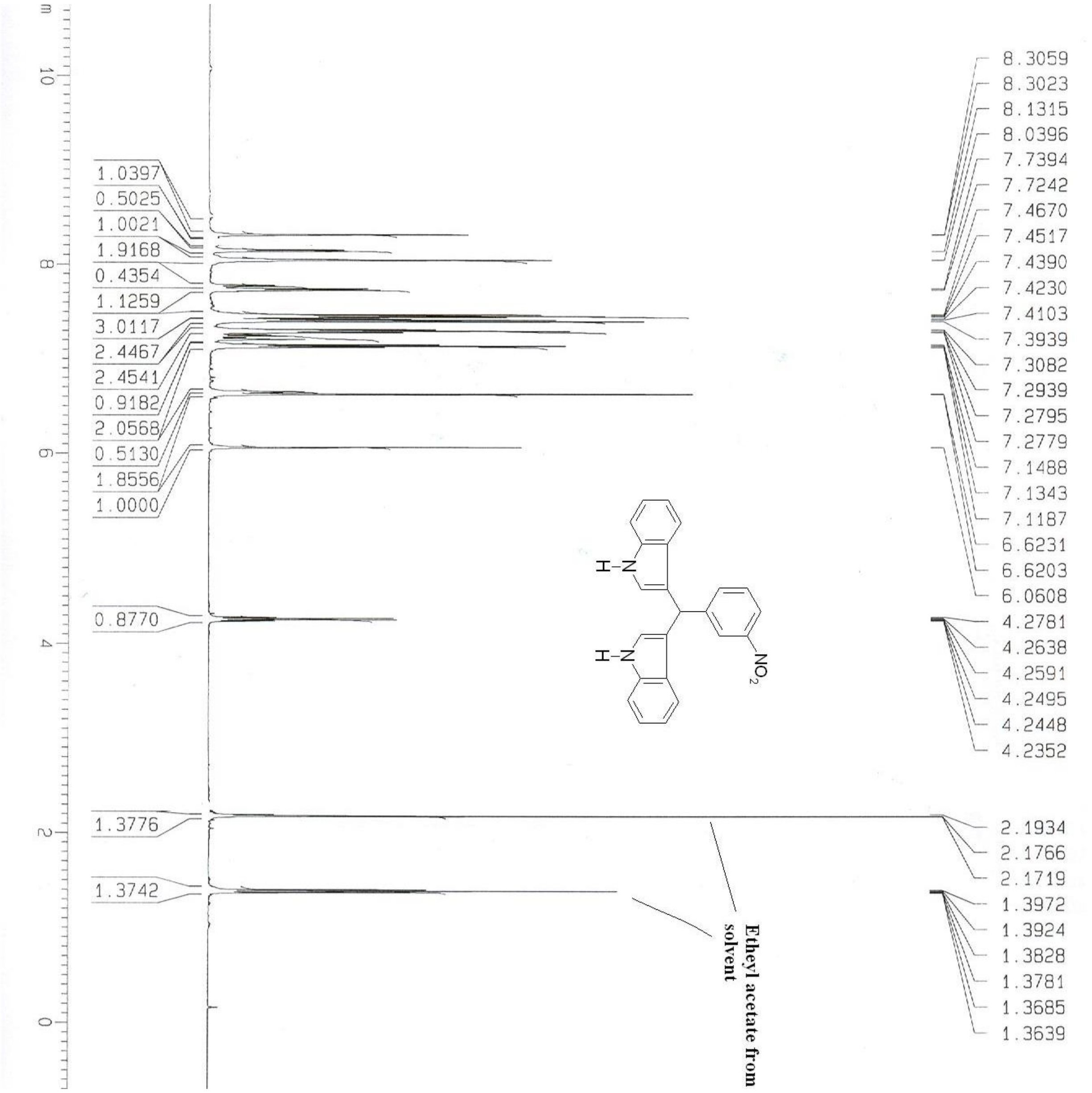


*Figure S 9. $^{1}$H NMR spectra of compound **3l** (Table 2, Entry 12).*

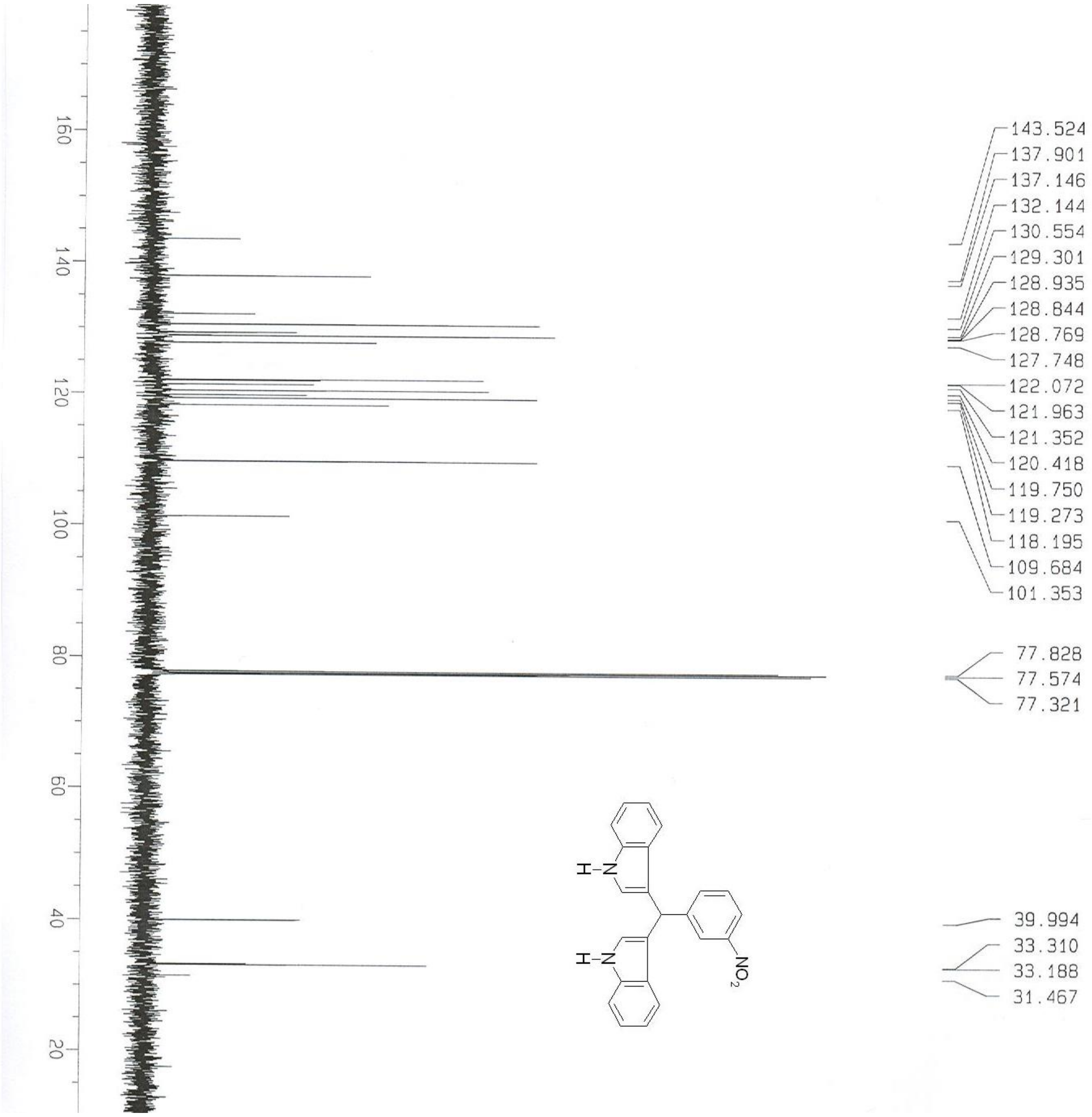


*Figure S 10. $^{13}$C NMR spectra of compound **3l** (Table 2, Entry 12).*

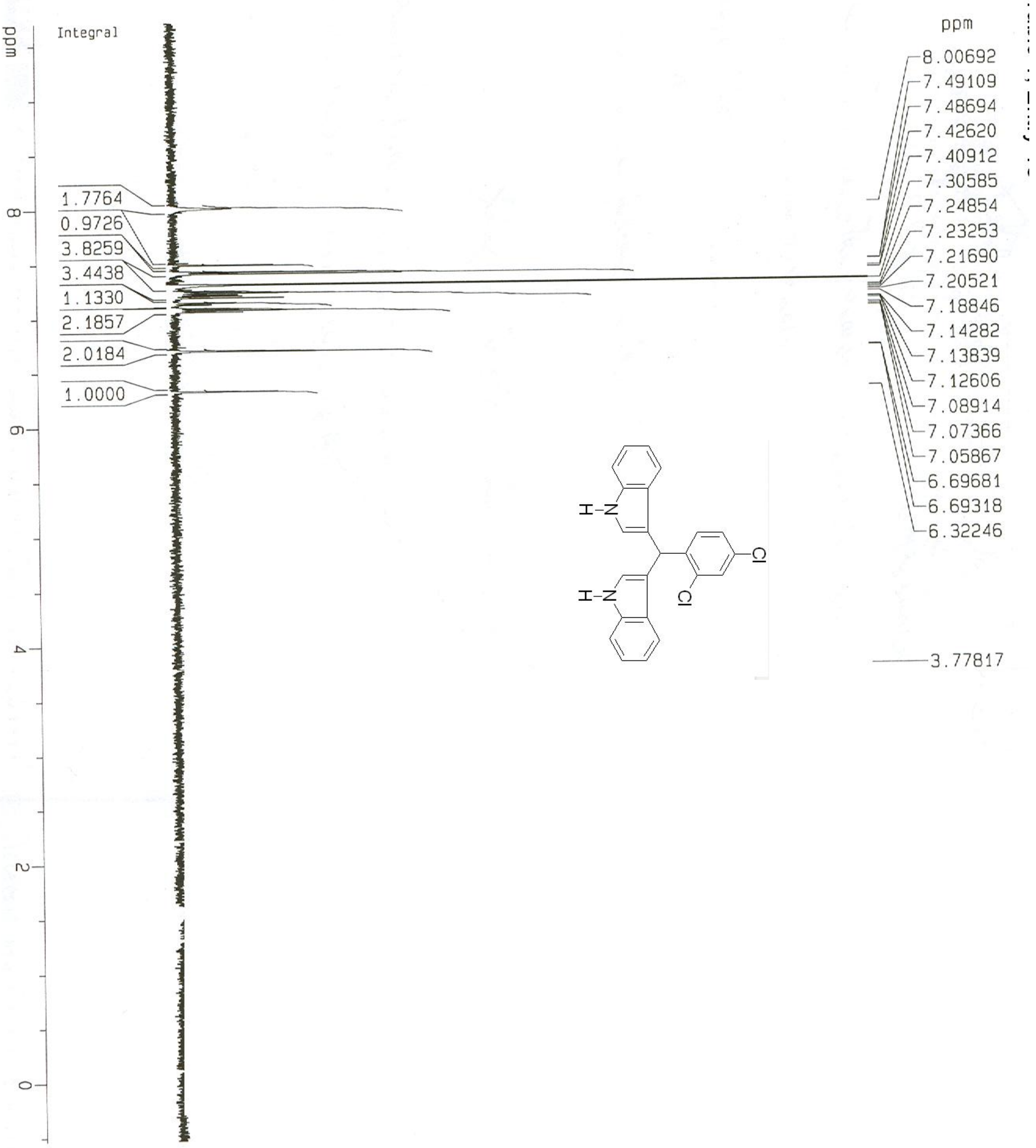


*Figure S 11. $^{1}$H NMR spectra of compound* **3m** *(Table 2, Entry 13).*

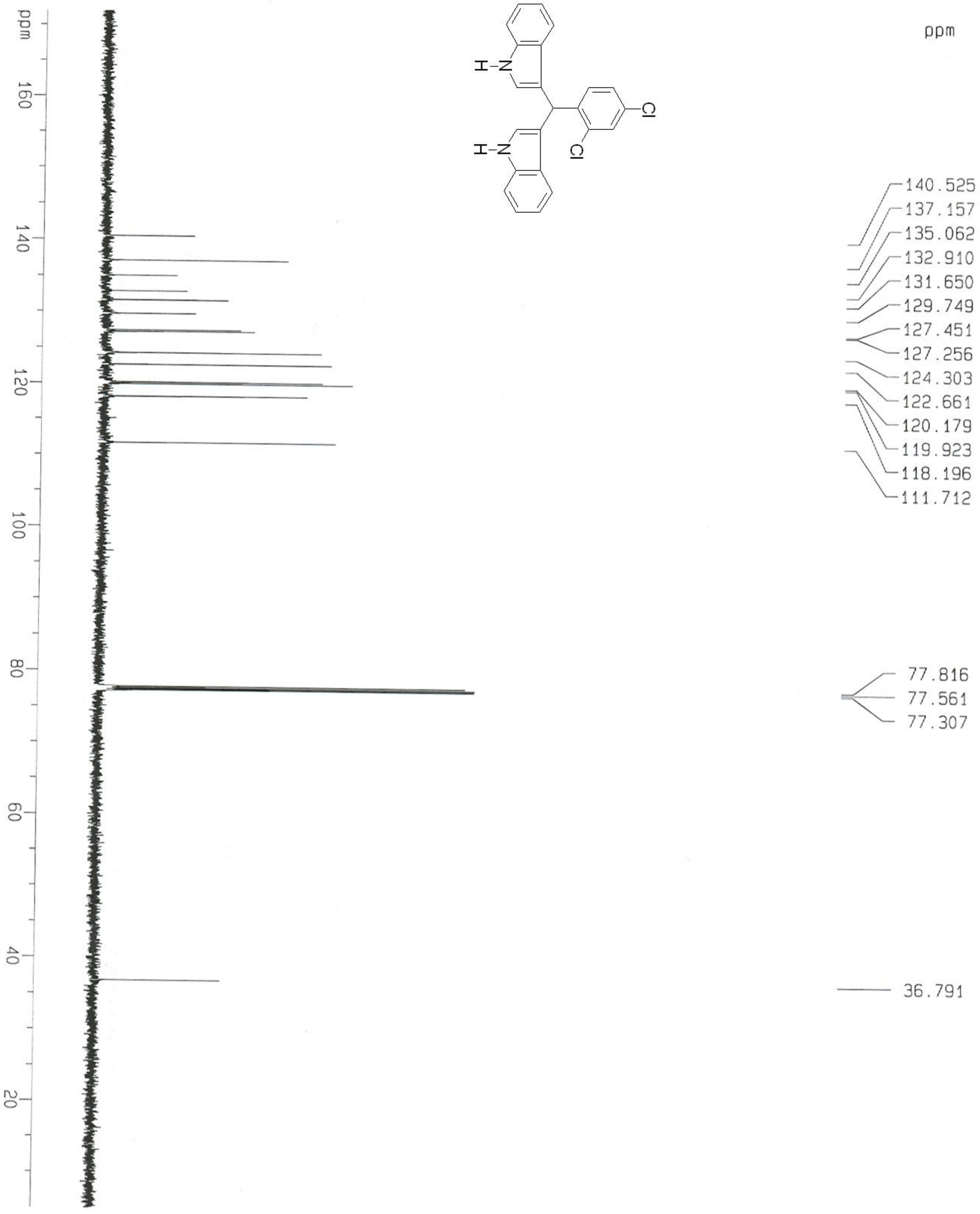


*Figure S 12. $^{13}$C NMR spectra of compound **3m** (Table 2, Entry 13).*

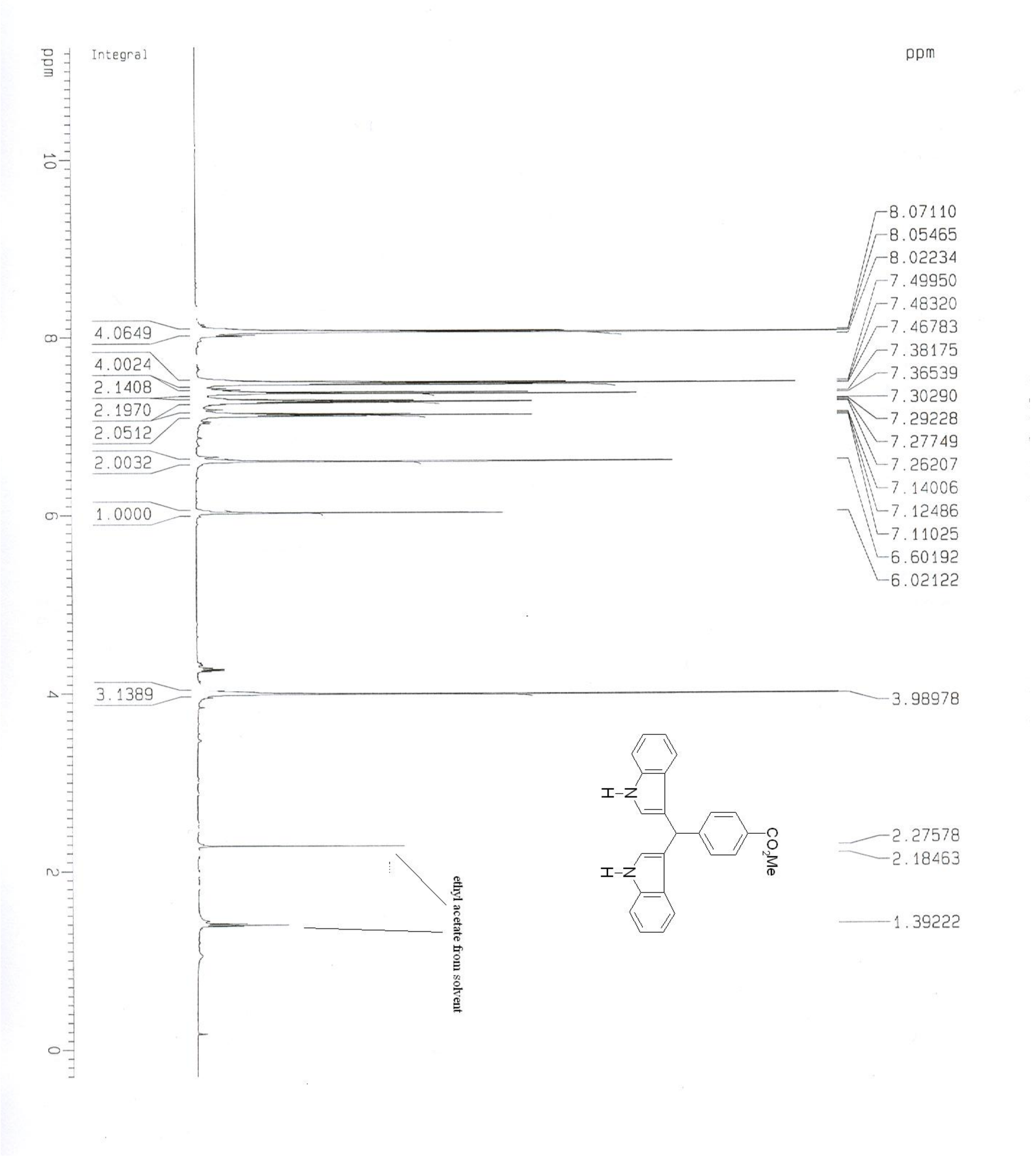


*Figure S 13. $^{1}$H NMR spectra of compound* ***3n*** *(Table 2, Entry 14).*

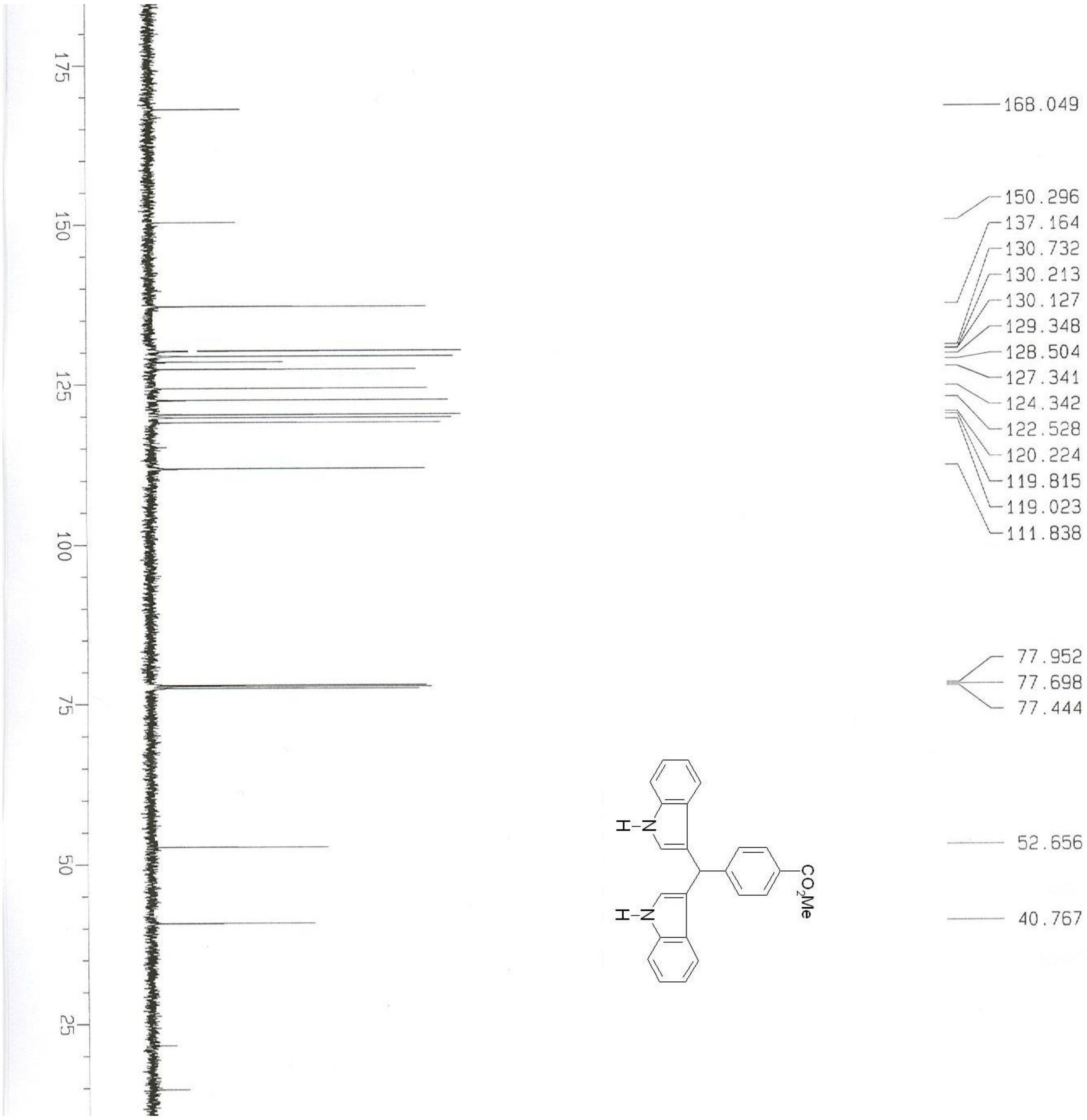


*Figure S 14. $^{13}$C NMR spectra of compound **3n**(Table 2, Entry 14).*

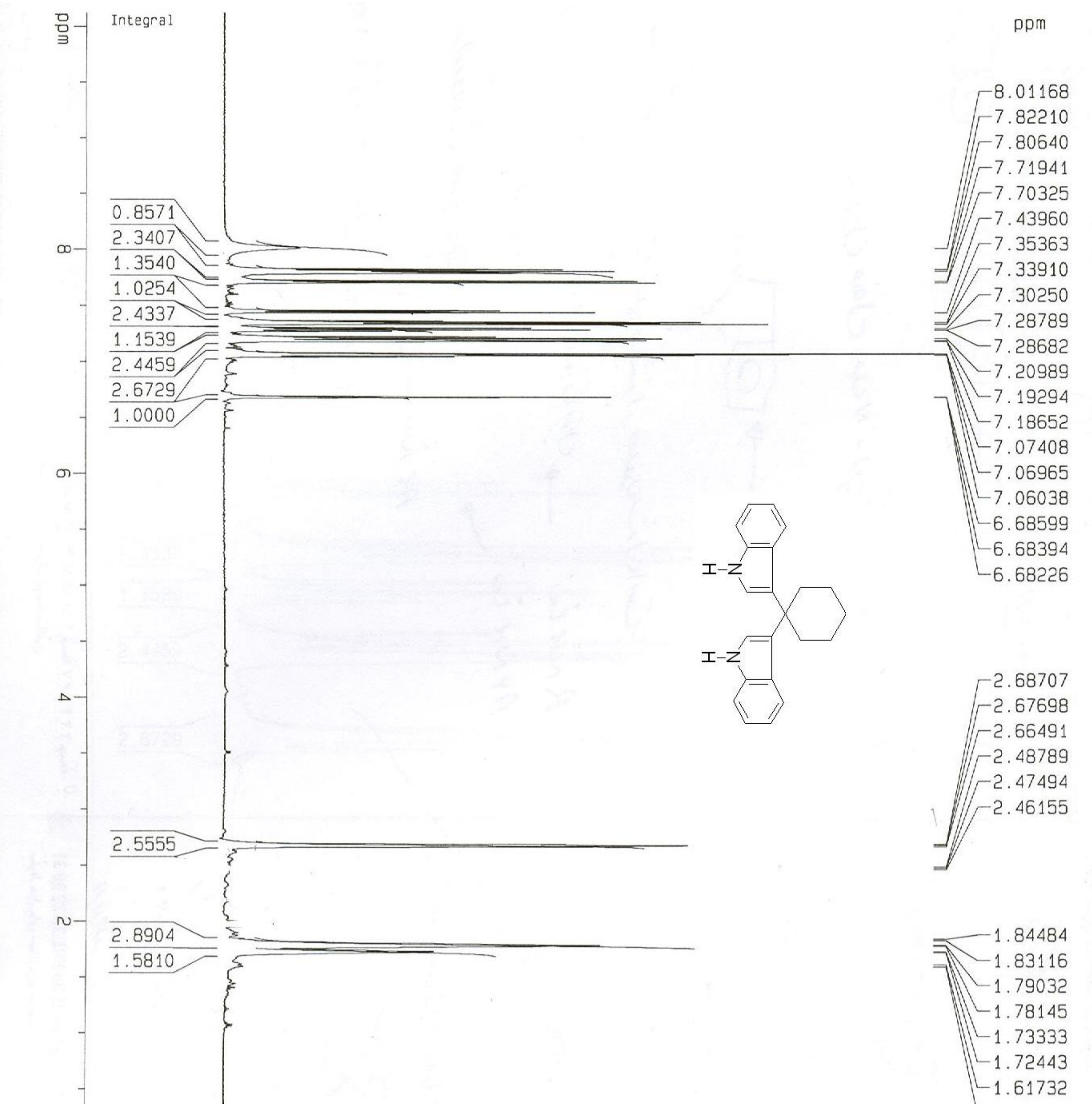


*Figure S 15. $^{1}$H NMR spectra of compound **3o** (Table 2, Entry 15).*

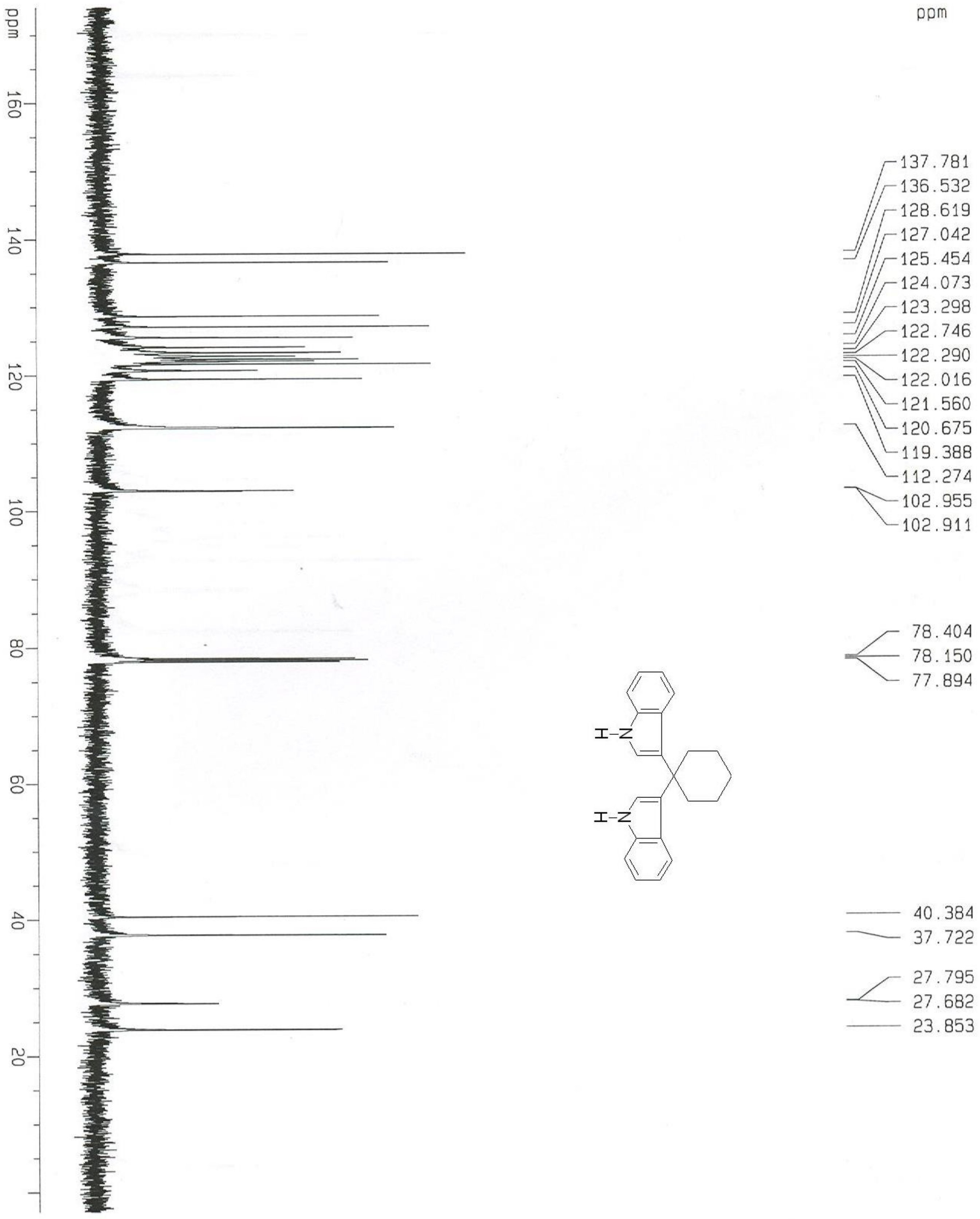


*Figure S 16. $^{13}$C NMR spectra of compound **3o** (Table 2, Entry 15).*
